# Effects of intracutaneous injections of sterile water in patients with acute low back pain: a randomized, controlled, clinical trial

**DOI:** 10.1590/1414-431X20155092

**Published:** 2016-02-02

**Authors:** J.Z. Cui, Z.S. Geng, Y.H. Zhang, J.Y. Feng, P. Zhu, X.B. Zhang

**Affiliations:** 1 Department of Pain Treatment, The First People’s Hospital of Lianyungang City, Lianyungang, Jiangsu Province, China; 2 Department of Anesthesiology, The First People’s Hospital of Lianyungang City, Lianyungang, Jiangsu Province, China

**Keywords:** Acute low back pain, Intracutaneous injection, Sterile water, Isotonic saline

## Abstract

Intracutaneous sterile water injection (ISWI) is used for relief of low back pain during labor, acute attacks of urolithiasis, chronic neck and shoulder pain following whiplash injuries, and chronic myofascial pain syndrome. We conducted a randomized, double-blinded, placebo-controlled trial to evaluate the effect of ISWI for relief of acute low back pain (aLBP). A total of 68 patients (41 females and 27 males) between 18 and 55 years old experiencing aLBP with moderate to severe pain (scores ≥5 on an 11-point visual analogue scale [VAS]) were recruited and randomly assigned to receive either ISWIs (n=34) or intracutaneous isotonic saline injections (placebo treatment; n=34). The primary outcome was improvement in pain intensity using the VAS at 10, 45, and 90 min and 1 day after treatment. The secondary outcome was functional improvement, which was assessed using the Patient-Specific Functional Scale (PSFS) 1 day after treatment. The mean VAS score was significantly lower in the ISWI group than in the control group at 10, 45, and 90 min, and 1 day after injection (P<0.05, *t*-test). The mean increment in PSFS score of the ISWI group was 2.9±2.2 1 day after treatment, while that in the control group was 0.9±2.2. Our study showed that ISWI was effective for relieving pain and improving function in aLBP patients at short-term follow-up. ISWI might be an alternative treatment for aLBP patients, especially in areas where medications are not available, as well as in specific patients (e.g., those who are pregnant or have asthma), who are unable to receive medications or other forms of analgesia because of side effects.

## Introduction

Low back pain is one of the most common problems presenting in primary care and is the most commonly reported type of pain worldwide (1 2 3 4 5). A total of 70-80% of adults have experienced at least one episode of acute low back pain (aLBP) or chronic low back pain in their lifetime (2,3). A total of 90% of patients with aLBP recover within 6 weeks (5). However, 2-7% of aLBP cases may develop chronic or persistent low back pain (3). Chronic low back pain is a major reason for workers to take paid sick leave and be absent from work, and it can result in early retirement with a disability pension in developed countries (4,6). Low back pain is associated with numerous adverse consequences, including prolonged loss of function, physical disability, loss of work productivity, psychosocial disruption, increased use of health care resources, and disability payments (4,7). Lack of effective and appropriate treatments for aLBP is one of the most common risk factors for developing chronic or persistent low back pain. Early, effective, and adequate management of aLBP is crucial for minimizing development of chronic or persistent low back pain.

The principal clinical goals of treatment for aLBP are to relieve pain, reduce time away from work, improve physical functioning, develop coping strategies through education, and diminish the likelihood of developing chronic low back pain. A wide range of pharmacological and non-pharmacological therapeutic treatments are available for aLBP, but their benefits and effectiveness still need to be verified (8 9 10). The most commonly prescribed medications for patients with moderate to severe aLBP include nonsteroidal anti-inflammatory drugs (NSAIDs), muscle relaxants, and opioids. However, there is little evidence demonstrating their durable therapeutic benefits (10 11 12). No substantial benefit has been indicated for acupuncture, oral steroids, massage, or lumbar support (9,13 14 15). Many patients may not have access to these therapies, or these therapies may be inapplicable because of side effects. Another reason for excluding regular pain management approaches is the strict safety requirements for some special populations. Pregnant women and patients with asthma cannot use certain pain medications. Therefore, a new treatment that is safe and effective without overt serious side effects is urgently required for such patients.

Several studies have demonstrated that intracutaneous sterile water injection (ISWI) provides statistically and clinically significant pain relief in women who experience continuous lower back pain during labor (16 17 18 19). This method has also been used to treat neck and shoulder pain in whiplash syndrome patients, cervicogenic headache, acute attacks of urolithiasis, and chronic myofascial pain syndrome (20 21 22 23 24 25). To the best of our knowledge, no experimental evidence is currently available to support the use of ISWI to treat aLBP, except for some reports of case studies (20,24).

Therefore, the present study aimed to determine whether ISWI is an effective method of ameliorating aLBP, especially for those who do not want, are unsuitable for, or do not have access to other pain therapies.

## Material and Methods

### Study design

This study was a randomized, placebo-controlled, double-blinded, clinical trial that evaluated the efficacy and safety of ISWI for the treatment of aLBP. This trial was conducted from March 2012 to February 2013. The randomization scheme was computer-generated and completed prior to the start of the study. The patients were randomly allocated to either the experimental group or control group at a ratio of 1:1. Experimental group patients received ISWI in the lumbosacral regions, while control group patients received corresponding intracutaneous injections of isotonic saline. We obtained the outcome variables at five different times: before treatment, at 10, 45, and 90 min, and 1 day post-treatment.

Written informed consent was obtained from each participant. The study was approved by the Ethics Committee of Lianyungang No.1 Hospital. We offered no economic incentives to the participants, and the patients were not billed for the treatment. The participants and treating clinician were blinded to treatment allocation.

### Participants

The patients (41 females and 27 males) included in this study were recruited from the First People’s Hospital of Lianyungang City, located in Jiangsu Province. The eligibility criteria for this trial were as follows: aLBP that was localized between the costal margin and above the inferior gluteal folds without radiating pain to the limb; aged between 18 and 50 years with aLBP of <2 weeks duration; the first episode of aLBP; and moderate to severe aLBP (scores ≥5 on an 11-point visual analogue scale [VAS]; 0="no pain", 10="worst conceivable pain"). Exclusion criteria were as follows: aLBP attributed to known or suspected serious pathology (e.g., inflammatory, infectious, or metastatic diseases of the spine, spinal fracture, spinal stenosis, osteoporosis, cauda equina syndrome, fibromyalgia); presumptive or confirmed lumbar nerve root compression; previous spinal surgery; pregnancy; patients who received any analgesic treatment within 12 h prior to recruitment in the study; experience of any side effects after taking NSAIDs; and reluctance or inability to complete the questionnaire.

### Sample size

The sample size was estimated using the mean difference in VAS scores for aLBP between the experimental and control groups. Based on previous pilot studies, the difference in the mean change in VAS score between the two groups was 2.2. We conservatively set this value as 2. When a two-tailed test with a test power of 80% and significance level of 5% was used, the minimum number of participants required for each group was 31 participants. To allow for 10% loss in follow-up, a total of 68 participants were required.

### Study intervention

The intracutaneous injection technique and examination of injection sites of all patients were conducted by the same pain specialist, who had >20 years of clinical experience. The injection sites consisted of all tender points (defined as feeling tender to pressure) and trigger points (defined as feeling tender and a radiating sensation when pressed). The location of each injection point was determined in accordance with previous studies and clinical experience (20,24,26). Tender points and trigger points were identified by digital palpation and marked with a ballpoint pen. These points were usually located over the lateral lumbar muscles, the margins of erector spinae and psoas muscles, and the lumbosacral area. Several patients also had tender points and trigger points along the anterior aspect of the lower half of the torso.

The patients were given intracutaneous injections of sterile water or isotonic saline at every injection point. A 2-mL plastic syringe (B. Braun Omnifix^¯^, Germany) with a thin needle (B. Braun Omnifix; diameter: 0.40 mm, length: 20 mm) was used for injections. After the needle was disinfected with alcohol, 0.5 mL sterile water or isotonic saline was injected to create blebs at the injection site. Three to five injections were administrated in rapid succession, emptying the syringe (i.e., 0.5 mL was injected at each point). The injections of sterile water caused a brief stinging sensation lasting for approximately 20 s (19). Therefore, a short break of 1 or 2 min was provided to allow any stinging sensation to fade after three to five injections. The injections were then continued until all points had been administrated. The patient then rested, lying down for 5-10 min. In both groups, after intracutaneous injections, an intramuscular injection of parecoxib sodium (40 mg; Pharmacia & Upjohn Company, USA) was administered intramuscularly in the gluteal region, if needed for additional treatment.

### Outcome measurements

Data on patients’ self-reported measures and data analysis were performed independently and strictly following the principle of double-blindedness. The primary outcome was pain intensity measured with the VAS (27), and this was recorded at baseline, at 10, 45, and 90 min, and 1 day post-treatment. The recorded secondary outcomes included patient-generated measurements of function, global rating of change, satisfaction with the intervention, whether they would accept the same treatment for a future episode of aLBP, the number of patients using intramuscular injection of parecoxib sodium for additional treatment, and adverse events.

The patient-generated measure of function was performed using the Patient-Specific Functional Scale (PSFS) at baseline and on the 1st day post-injection (28,29). The PSFS (0-10 scale; 0="unable to perform activity", 10="able to perform activity at the same level as before the injury or problem") is a patient-specific outcome measurement that examines functional status. Patients were required to nominate five activities with which they had difficulties because of pain and then rate the functional limitation related to these activities.

One day after treatment, the patients were asked to rate their global rating of change on a 7-point Likert scale with responses of 1="completely gone", 2="much better", 3="better", 4="a little better", 5="about the same", 6="a little worse", and 7="much worse" (30,31). The patients were also asked to rate their satisfaction with intervention on an 11-point scale, from 0="not at all satisfied" to 10="extremely satisfied" (31).

The number of participants using intramuscular injections of parecoxib sodium for additional treatment and the number of adverse events at day 1 post-injection were recorded. On the first post-injection day, the patients were asked to complete a questionnaire regarding whether they would accept the same treatment during a future episode of aLBP.

### Statistical analysis

Continuous variables were compared using the independent *t*-test and they are reported as means±SD. Categorical variables were compared using the chi-square test or Fisher’s exact test and are reported as numbers or percentages. Furthermore, the Mann-Whitney U test was used if the normality assumption was violated. For comparison of the VAS, PSFS, global rating of change, and satisfaction with intervention between the two groups, the independent *t*-test or Mann-Whitney U test was used. The percentage of participants using intramuscular injection of parecoxib sodium for additional treatment, the percentage of adverse events, and the percentage of participants who accepted the same treatment during a future episode of aLBP in each group were calculated and compared using the chi-square test or Fisher’s exact test. Two-tailed tests at a significance level of 0.05 were used. All statistical analyses were performed using SPSS version 16.0 software (IBM Corporation, USA).

## Results

### Screening, enrollment, and follow-up

**Figure 1:**
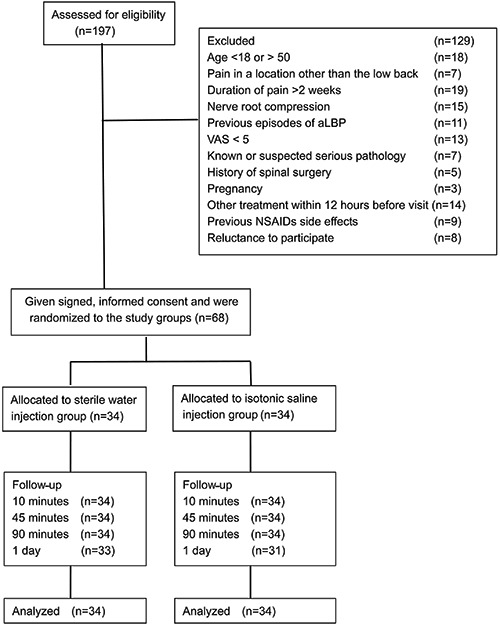
Study flow chart.

A flowchart of the study is shown in Figure 1. A total of 197 potential participants were screened for the study and 68 were enrolled. Randomization resulted in 34 participants assigned to each group. All 68 participants were evaluated at 10, 45, and 90 min of post-treatment follow-up. A total of 33 participants in the experimental group and 31 in the control group answered the entire follow-up questionnaire on the first day after treatment. The main reason for loss to follow-up was being unable to make contact with the participants.

### Demographic data

Table 1 shows the demographic data of the patients and their baseline characteristics. The mean age of the study participants was 32.3±8.5 years. The mean body mass index (BMI) was 24.2±2.5 kg/m^2^. A total of 41 of 68 (60.1%) patients were women. A total of 29 of 68 (42.6%) patients claimed to perform regular physical exercise, and 40 of 68 (58.8%) patients were married. No significant differences in age, BMI, sex, regular physical exercise, and marital status were observed between the two groups. At baseline, the mean rating of pain intensity score was 6.7 for the experimental group and 6.6 for the control group, with no difference between the groups (P=0.919). The median duration of current aLBP episodes in participants at the time of enrollment was 5.4 days. There was no significant difference in the PSFS score and the number of injection sites between the two groups (Table 1).

**Table:**
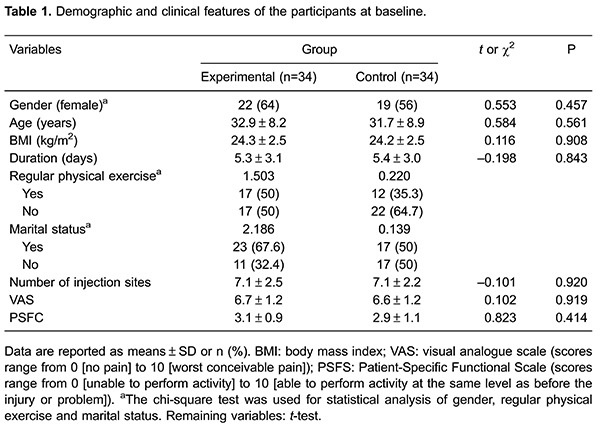

### Primary outcome

Ten minutes after treatment, the experimental group had a significantly lower mean VAS score (2.7±1.9 points) than the control group (4.9±1.2 points; P<0.001). This significant difference in the VAS was maintained at all other follow-up examination times (Table 2).

**Table:**
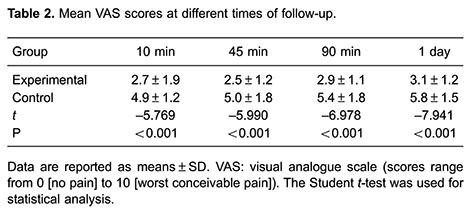

There was a significant reduction in VAS score at 10 min after treatment compared with that at baseline in both groups, but this difference was more pronounced in the experimental group (P<0.001). The mean VAS score was also significantly more reduced in the experimental group compared with the control group at 45 min (P<0.001), 90 min (P<0.001), and 1 day post-treatment (P=0.029). The mean difference in the pre- and post (10 min)-injection VAS score between the two groups was 2.2 points (95% confidence interval: 1.4-3.0; P<0.001) in favor of the experimental group. This finding indicated that, on average, ISWI induced a greater reduction in pain than did intracutaneous injections of isotonic saline. This difference was also observed at 45 min, 90 min, and 1 day post-treatment (Table 3).

**Table:**
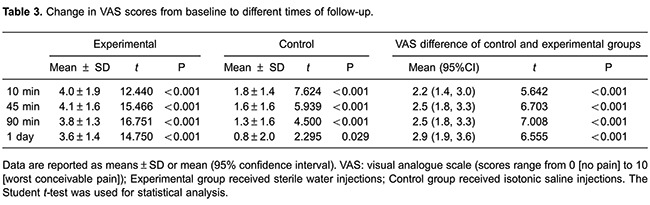

### Secondary outcome

The mean PSFS 1 day after treatment was significantly higher than the pretreatment score (P<0.001) in both groups. The PSFS score 1 day after treatment in the experimental group (6.2±1.8) was significantly higher compared with that in the control group (3.8±2.0; P<0.001). The mean increment in PSFS score on a 0 to 10 scale in the experimental group was 2.9±2.2 1 day after treatment, while the mean increment in the control group was 0.9±2.2. The mean difference in increment of the PSFS score between the two groups was 2.0 points in favor of the experimental group (95% confidence interval: 1.0-3.2; P<0.001).

For the patients’ global impression (global rating of change) of the treatment, there was a significant difference between the two groups as follows. The experimental group showed a higher level of satisfaction with the treatment (6.6±2.0) than did the control group (3.6±2.5; P<0.001; Table 4).

**Table:**
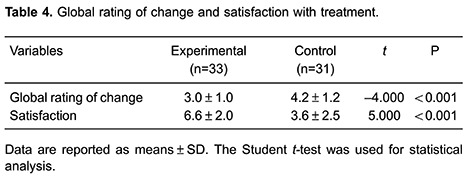

The percentage of the experimental group (9%, 3/33) using parecoxib for their additional pain relief subsequent to sterile water injection was not significantly different from that in the control group (19%, 6/31; P=0.238). More patients in the experimental group (64%) claimed that they would want to use the same pain relief method in a future episode of aLBP than those in the control group (35%; P=0.024).

No serious adverse events were observed during the trial in either group. The only complaint reported in the trial was transient burning pain at the injection sites, and this pain lasted approximately 20–30 s. A higher percentage of patients in the experimental group (65%, 22/34) reported transient burning pain than did the control group (35%, 12/34; P=0.015).

## Discussion

This study shows the safety and efficacy of ISWI versus placebo (isotonic saline) in patients with aLBP. ISWI provided a higher degree of pain relief and functional improvement than did placebo injections. These results suggest that ISWI has superior effects on reduction of pain and improvement in functional status, as shown by the VAS scores of aLBP and PSFS scores, compared with isotonic saline injection.

Our study also indicated that intracutaneous isotonic saline injections had a significant effect in reducing pain and increasing function. This finding is in agreement with previous observations during pain research in which placebo management had a considerable analgesic potency (19,26). The reason for this finding is unclear, but it might be related to the fact that ISWI causes osmotic stimulation and dilation of compact layers of the skin, whereas isotonic saline injection can only cause inflation in the compact layers of the skin (32). Placebo management is antagonized by naloxone (33), which supports the theory that the placebo effect in pain management is, at least to some degree, mediated by endogenous opioids. Furthermore, the potential beneficial effect of sterile water treatment may have been underestimated because it should have been compared with a true placebo.

In aLBP, sometimes there is a brief "hyperacute" period of 24-48 h during which patients are essentially immobilized and motion is hampered by pain and intense spasm. Fortunately, this hyperacute period occurs in a small amount of patients and generally resolves within 24-48 h (10). In our study, the onset of an aLBP episode in a small number of patients was within 48 h. Therefore, we were unable to evaluate the degree to which there was a significant improvement in pain intensity and function for both groups because of the natural characteristics of aLBP progression. Additionally, we did not attempt to restrict all medication treatments because of ethical reasons in both groups. However, there was no significant difference in the number of patients who managed their pain using medication (parecoxib) after the initial treatment between the two groups. This finding suggested that medication use was unlikely to be a confounding factor of the results.

As previously reported, transient intense pain associated with administration negatively affects patients’ experiences of ISWI (16,17,19,20,22). In our study, patients in the experimental group, with a greater difference in pre- and post-injections scores than the control group, were more likely to rate their experience positively, regardless of the perceived injection pain. Previous studies have established that there is a relationship between patients who found sterile water injections effective or ineffective, and those who would or would not accept the procedure again (16,32). If the administration pain itself is an overt negative factor in patients’ experience of sterile water injection, there is likely to be disparity between the rating scores of pain intensity and those likely to use it again in the future. This suggests that people will accept the pain associated with ISWI if there is an analgesic effect. Conversely, administration pain is likely to influence the rating scores of satisfaction when the procedure is perceived as ineffective. Notably, our study was performed in patients with various special clinical features, and this group may be different to the whole patient population that has aLBP. Therefore, these results may not be directly extrapolated to other types of patients.

Recently reported clinical trials have focused on the practicality of ISWI for different pain syndromes. However, the results of these trials are not concordant. In accordance with the results of pain in our study, ISWI has been reported to relieve acute labor pain, acute renal colic pain, neck and shoulder pain in whiplash syndrome, and chronic myofascial pain syndrome (20 21 22 23 24 25). However, Sand et al. (21) did not find any effects of ISWI on pain intensity and neck mobility in patients with cervicogenic headache. The reasons for this difference between studies are unclear, but some possibilities include elements of the different nature of pain, different numbers and sites of injection, different pain mechanisms in patients, or some other unknown reasons. In the current study, we did not attempt to determine the mechanism of pain relief by sterile water injection. Different theories, such as the classical gate control mechanism, hyperstimulation or counter-irritation, physiological distraction, and diffuse noxious inhibitory control, have been proposed to explain the mechanism of action of this method (18,34 35 36). Another explanation is that ISWI may lead to endogenous opioid release, similar to that observed with transcutaneous electrical nerve stimulation or acupuncture (37,38).

An important strength of this study is that interventions were performed by the same experienced clinician who was blind to the group allocation and outcome measures. Additionally, the participants and the outcome investigator and analyst remained blinded to treatment allocation throughout the study. A further strength of our study was the small overall loss to follow-up (<5%).

The primary limitation of this study was the fact that we did not include a control group without treatment. Therefore, we cannot make conclusions about "absolute" treatment effectiveness for ISWI or isotonic saline. Clinically significant results may differ from statistically significant results. Previous studies have reported that a reduction in VAS score of ≥3.5 and improvement in PSFS score of ≥2 are clinically significant in low back pain patients (28,39). In our study, the ISWI group showed a reduction in VAS score of ≥3.5 at all follow-up times and an increase in PSFS score of 2.9±2.2 1 day after treatment. These results are clinically significant levels of reduction in pain and functional improvement. Another limitation was that our study included only self-reported measures of pain intensity, and improvements in function, both of which are subjective. More objective measurements may have generated a different result. Moreover, assessment of the effect of this method should focus on improvements in pain, function, and mood simultaneously. Other limitations include rigorous entry criteria for aLBP, no intermediate-term and long-term follow-up, and the patients’ expectations and the successfulness of the blinding attempts were not assessed. Finally, the source of aLBP was likely to vary among patients because aLBP has a high degree of heterogeneity and intrinsic variability.

In future studies, the maximum duration of pain relief and the effectiveness of repeated injections of sterile water in patients with aLBP should be determined. The optimal tissue depth for injection and the volume of sterile water to be injected should also be precisely defined. The use of subcutaneous injections to reduce the intense burning pain associated with intracutaneous injections and evaluation of the effects of subcutaneous sterile water injections on aLBP could be considered for future studies. Finally, future studies should also establish the treatment effect of ISWIs on aLBP in actual clinical practice. Currently, there are few data to support whether a subgroup of individuals are more likely to benefit from this method. Therefore, stringent eligibility criteria of the study may be an important consideration for future clinical applications.

Our data showed that ISWI was effective in ameliorating pain and improving function in aLBP patients. This procedure is safe, easy to perform, inexpensive, and suitable for almost everyone. This method may be suitable for patients with moderate to severe aLBP including those who are too old or too young for other treatments, who may not have access to medications, those who are purposely trying to avoid other forms of analgesia because of perceived or actual side effects, and those in rural and remote areas or in some developing countries.

## References

[1] Hoy D, Brooks P, Blyth F, Buchbinder R (2010). The Epidemiology of low back pain. Best Pract Res Clin Rheumatol.

[2] Freburger JK, Holmes GM, Agans RP, Jackman AM, Darter JD, Wallace AS (2009). The rising prevalence of chronic low back
pain. Arch Intern Med.

[3] Rubin DI (2007). Epidemiology and risk factors for spine
pain. Neurol Clin.

[4] Parthan A, Evans CJ, Le K (2006). Chronic low back pain: epidemiology, economic burden and
patient-reported outcomes in the USA. Expert Rev Pharmacoecon Outcomes Res.

[5] van Tulder M, Becker A, Bekkering T, Breen A, del Real MT, Hutchinson A (2006). Chapter 3. European guidelines for the management of
acute nonspecific low back pain in primary care. Eur Spine J.

[6] Maetzel A, Li L (2002). The economic burden of low back pain: a review of
studies published between 1996 and 2001. Best Pract Res Clin Rheumatol.

[7] Linton SJ (1998). The socioeconomic impact of chronic back pain: is anyone
benefiting?. Pain.

[8] Abdel Shaheed C, Maher CG, Williams KA, McLachlan AJ (2014). Interventions available over the counter and advice for
acute low back pain: systematic review and meta-analysis. J Pain.

[9] Lee JH, Choi TY, Lee MS, Lee H, Shin BC, Lee H (2013). Acupuncture for acute low back pain: a systematic
review. Clin J Pain.

[10] Kuritzky L, Samraj GP (2012). Nonsteroidal anti-inflammatory drugs in the treatment of
low back pain. J Pain Res.

[11] Cifuentes M, Webster B, Genevay S, Pransky G (2010). The course of opioid prescribing for a new episode of
disabling low back pain: opioid features and dose escalation. Pain.

[12] van Tulder MW, Touray T, Furlan AD, Solway S, Bouter LM (2003). Muscle relaxants for nonspecific low back pain: a
systematic review within the framework of the cochrane
collaboration. Spine.

[13] Holve RL, Barkan H (2008). Oral steroids in initial treatment of acute
sciatica. J Am Board Fam Med.

[14] van Duijvenbode I, Jellema P, van Poppel MN, van Tulder MW (2008). Lumbar supports for prevention and treatment of low back
pain. Cochrane Database Syst Rev.

[15] Furlan AD, Imamura M, Dryden T, Irvin E (2009). Massage for low back pain: an updated systematic review
within the framework of the Cochrane Back Review Group. Spine.

[16] Lee N, Webster J, Beckmann M, Gibbons K, Smith T, Stapleton H (2013). Comparison of a single *vs* a four
intradermal sterile water injection for relief of lower back pain for women in
labour: a randomised controlled trial. Midwifery.

[17] Lee N, Coxeter P, Beckmann M, Webster J, Wright V, Smith T (2011). A randomised non-inferiority controlled trial of a
single versus a four intradermal sterile water injection technique for relief of
continuous lower back pain during labour. BMC Pregnancy Childbirth.

[18] Derry S, Straube S, Moore RA, Hancock H, Collins SL (2012). Intracutaneous or subcutaneous sterile water injection
compared with blinded controls for pain management in labour. Cochrane Database Syst Rev.

[19] Ader L, Hansson B, Wallin G (1990). Parturition pain treated by intracutaneous injections of
sterile water. Pain.

[20] Byrn C, Olsson I, Falkheden L, Lindh M, Hosterey U, Fogelberg M (1993). Subcutaneous sterile water injections for chronic neck
and shoulder pain following whiplash injuries. Lancet.

[21] Sand T, Bovim G, Helde G (1992). Intracutaneous sterile water injections do not relieve
pain in cervicogenic headache. Acta Neurol Scand.

[22] Xue P, Tu C, Wang K, Wang X, Fang Y (2013). Intracutaneous sterile water injection versus oral
paracetamol for renal colic during pregnancy: a randomized controlled
trial. Int Urol Nephrol.

[23] Ahmadnia H, Younesi RM (2004). Treatment of renal colic using intracutaneous injection
of sterile water. Urol J.

[24] Byrn C, Borenstein P, Linder LE (1991). Treatment of neck and shoulder pain in whip-lash
syndrome patients with intracutaneous sterile water injections. Acta Anaesthesiol Scand.

[25] Wreje U, Brorsson B (1995). A multicenter randomized controlled trial of injections
of sterile water and saline for chronic myofascial pain syndromes. Pain.

[26] Loughnan TE, Taverner MG, Webb A (2009). Randomized, double blinded comparative trial of
intradermal injections of lignocaine versus N-saline around the knee to relieve
pain in patients awaiting total knee replacement. Clin J Pain.

[27] Price DD, Bush FM, Long S, Harkins SW (1994). A comparison of pain measurement characteristics of
mechanical visual analogue and simple numerical rating scales. Pain.

[28] Stratford PW, Binkley J (1995). The Quebec Back Pain Disability Scale: measurement
properties. Spine.

[29] Pengel LH, Refshauge KM, Maher CG (2004). Responsiveness of pain, disability, and physical
impairment outcomes in patients with low back pain. Spine.

[30] Patrick DL, Deyo RA, Atlas SJ, Singer DE, Chapin A, Keller RB (1995). Assessing health-related quality of life in patients
with sciatica. Spine.

[31] Goertz CM, Long CR, Hondras MA, Petri R, Delgado R, Lawrence DJ (2013). Adding chiropractic manipulative therapy to standard
medical care for patients with acute low back pain: results of a pragmatic
randomized comparative effectiveness study. Spine.

[32] Martensson L, Wallin G (1999). Labour pain treated with cutaneous injections of sterile
water: a randomised controlled trial. Br J Obstet Gynaecol.

[33] Levine JD, Gordon NC (1984). Influence of the method of drug administration on
analgesic response. Nature.

[34] Melzack R, Wall PD (1965). Pain mechanisms: a new theory. Science.

[35] Melzack R (1975). Prolonged relief of pain by brief, intense
transcutaneous somatic stimulation. Pain.

[36] Morgan MM, Whitney PK (1996). Behavioral analysis of diffuse noxious inhibitory
controls (DNIC): antinociception and escape reactions. Pain.

[37] Terenius L, Tamsen A (1982). Endorphins and the modulation of acute
pain. Acta Anaesthesiol Scand Suppl.

[38] Chapman CR, Benedetti C (1977). Analgesia following transcutaneous electrical
stimulation and its partial reversal by a narcotic antagonist. Life Sci.

[39] Ostelo RW, de Vet HC (2005). Clinically important outcomes in low back
pain. Best Pract Res Clin Rheumatol.

